# Aromatase and CDK4/6 Inhibitor-Induced Musculoskeletal Symptoms: A Systematic Review

**DOI:** 10.3390/cancers13030465

**Published:** 2021-01-26

**Authors:** Angeliki Andrikopoulou, Oraianthi Fiste, Michalis Liontos, Meletios-Athanasios Dimopoulos, Flora Zagouri

**Affiliations:** 1 Department of Clinical Therapeutics, Alexandra Hospital, Medical School, 11528 Athens, Greece; aggandrikop@med.uoa.gr (A.A.); ofiste@med.uoa.gr (O.F.); mlionto@med.uoa.gr (M.L.); mdimop@med.uoa.gr (M.-A.D.); 2 Medical School, National and Kapodistrian University of Athens, 80 Vasilissis Sofias Avenue, 11528 Athens, Greece

**Keywords:** breast, cancer, aromatase, CDK4/6 inhibitors, arthralgia, musculoskeletal

## Abstract

**Simple Summary:**

Aromatase inhibitor-induced musculoskeletal symptoms (AIMSS) occurs in up 50% of postmenopausal patients and is the reason for treatment discontinuation in 25% of patients with breast cancer. CDK4/6 inhibitors have been established in the treatment of hormone receptor-positive (HR) breast cancer. We aimed to assess the effect of treatment with CDK4/6 inhibitors on AIMSS. Arthralgia rate was lower in patients receiving aromatase inhibitors (AIs) in combination with CDK4/6 inhibitors (5.8–33.3%) compared with monotherapy with AIs (1–47%). Myalgias, back pain and bone pain also tended to be reduced in patients treated with CDK4/6 inhibitors. CDK4/6 inhibitors may alleviate musculoskeletal pain caused by AIs, although further studies are warranted.

**Abstract:**

Background: Treatment with aromatase inhibitors (AIs) is fundamental in women with hormone receptor-positive breast cancer in the adjuvant as well as the metastatic setting. Even though it is considered to be a well-tolerated therapy, aromatase inhibitor-associated musculoskeletal syndrome (AIMSS) is the most common adverse event encountered by breast cancer patients. CDK4/6 inhibitors have emerged as a new treatment strategy in metastatic hormone receptor-positive breast cancer. However, the impact of CDK4/6 inhibitors on musculoskeletal symptoms caused by AIs is not well-defined. Objectives: This systematic review aims to identify the frequency of joint symptoms induced by treatment with AIs and CDK4/6 inhibitors in the metastatic setting. Search strategy: Eligible articles were identified by a search of existing literature for the period 2005/01/01–2021/01/01; The algorithm consisted of a predefined combination of the following keywords “breast”, “cancer”, “aromatase inhibitors”, “CDK4/6”, “phase III”. Selection criteria: This study was performed in accordance with PRISMA guidelines. All randomized controlled Phase III trials (RCTs) evaluating the administration of third-generation aromatase inhibitors (AIs) and CDK4/6 inhibitors in postmenopausal women in the metastatic setting were considered eligible for this review. Data collection: Overall, 16 randomized control trials (RCTs) were retrieved, of which nine studies explored the administration of AIs in the metastatic setting and seven studies investigated the combination of CDK4/6 inhibitors and AIs. Arthralgia was reported in 1–47% of patients treated with AIs and 5.8–33.3% of patients treated with CDK4/6 inhibitors. Myalgias occurred in 2–23.7% of patients receiving AIs compared with 4.8–11.9% of patients treated with CDK4/6 inhibitors. The incidence of back pain was 7–32.9% vs. 2.9–8.5% in postmenopausal women with metastatic disease treated with AIs and CDK4/6 inhibitors, respectively. Bone pain was reported in 7–32.9% of postmenopausal women treated with AIs and 2.9–8.5% of women treated with CDK4/6 inhibitors. Conclusions: AI treatment-induced musculoskeletal syndrome is an adverse event affecting over one-third (20–47%) of postmenopausal patients treated with AIs that often leads to treatment discontinuation. Data from RCTs provide evidence that the incidence of musculoskeletal symptoms is relatively decreased upon CDK4/6 inhibitor administration. CDK4/6 inhibitors may provide a protective role against AIMSS development.

## 1. Introduction

Breast cancer is the most commonly diagnosed cancer type in women in both developed and developing countries. It is estimated that in 2020, 276,480 new cases and 42,170 new deaths will be reported in the United States, making breast cancer the second leading cause of cancer-related mortality in women [1]. Around 75% of all breast cancer cases express the estrogen receptor (ER) and/or the progesterone receptor (PgR) and are considered hormone receptor-positive (HR) tumors [2]. Women who present with early hormone receptor-positive breast cancer are treated with surgery and adjuvant endocrine therapy (ET), but metastatic disease eventually develops in up to 40% despite adjuvant treatment [3]. In addition, almost two-thirds of de novo metastatic breast cancer cases are hormone receptor-positive [4]. Consequently, endocrine therapy is the cornerstone of H-positive disease treatment in both the adjuvant and metastatic setting. Third-generation aromatase inhibitors (AI), including letrozole, anastrozole and exemestane, are well established in the endocrine therapy of postmenopausal breast cancer patients. The recommended treatment approach for women in the adjuvant setting is aromatase inhibitor administration for a total of 5–10 years [5] as there are several trials confirming their superiority over treatment with tamoxifen [6,7]. However, despite the initial response to endocrine therapy, 25% of H-positive early-stage breast cancer cases and almost all metastatic cases eventually develop resistance to treatment [8]. Currently, CDK4/6 inhibitors have emerged as new treatment options in the management of metastatic H-positive breast cancer in combination with aromatase inhibitors [9,10,11]. CDK4/6 inhibition attenuates CDK4/6-mediated phosphorylation of retinoblastoma tumor-suppressor protein (RB) and thus maintains RB-E2F interactions leading to G1 cell cycle arrest [12].

Even though therapy with AI is considered to be well-tolerated, patients receiving AI tend to experience adverse events very often. Aromatase inhibitor-induced arthralgia is estimated to occur in up 50% of postmenopausal patients, according to a recent meta-analysis [13]. Aromatase inhibitor-induced musculoskeletal symptoms (AIMSS) may present with arthralgia, myalgia, arthritis or stiffness, and it may be the reason for discontinuation of the treatment in approximately 25% of the patients with early breast cancer [14]. In addition, treatment with AI resulted in a bone loss rate at least two-fold higher than age-matched postmenopausal women increases the risk of fractures [15]. Symptoms typically occur in the upper and lower extremities, mainly in hands and wrists, ankles, shoulders and knees [16]. Given the established benefit of AI treatment in H-positive breast cancer, it is essential to early recognize and better understand aromatase inhibitor-associated musculoskeletal syndrome as well as identify the prevalence of this adverse event. This systematic review summarizes all available data concerning musculoskeletal symptoms reported in randomized Phase III trials evaluating the administration of aromatase inhibitors as a monotherapy or in combination with CDK4/6 inhibitors in the metastatic setting. We aim to clarify the incidence of AIMSS in AI monotherapy treatment in postmenopausal women with metastatic disease and the impact of the recent addition of CDK4/6 inhibitors on this common adverse event.

## 2. Materials and Methods

### 2.1. Search Strategy and Eligibility of Studies

This systematic review was performed in accordance with PRISMA guidelines [17]. The protocol of this systematic review was submitted to the Institutional Review Board of Alexandra Hospital and Medical School of Athens and is available upon request. Eligible studies were all randomized controlled trials (RCTs) identified using MEDLINE bibliographical and ClinicalTrials.gov database for search concerning the period 2005/01/01–2021/01/01 so as not to confound data with the combination of AIs and the progestin megestrol acetate that used to be given until then [18,19,20,21,22] or other AIs, e.g., formestane [23]. The search algorithm applied consisted of the following words: (breast AND (cancer OR neoplasm) and (aromatase inhibitors OR letrozole OR anastrozole OR exemestane OR CDK4/6 OR palbociclib OR ribociclib OR abemaciclib) AND (phase III). Language restrictions were not applied. In order to maximize the amount of synthesized information, we systematically examined the reference lists of the articles retrieved for potentially eligible studies.

Eligible studies included all randomized controlled Phase III trials exploring third-generation AI monotherapy treatment in postmenopausal women with locally advanced/metastatic breast cancer and all randomized controlled Phase III trials evaluating AIs in combination with CDK4/6 inhibitors in postmenopausal patients with advanced or metastatic breast cancer or premenopausal patients that underwent ovarian suppression with GnRH analog, e.g., MONALEESA-7 [24,25]. Phase I-II trials, case reports and reviews were excluded, e.g., FIRST trial [26]. Phase III trials exploring CDK4/6 inhibitors in metastatic breast cancer were considered eligible only when administered in combination with aromatase inhibitors. In addition, trials of AIs administered in combination with other antineoplastic drugs, e.g., trastuzumab, everolimus [27,28,29,30,31,32,33] or in combination with bone-protective therapies [34,35,36,37], were excluded. Retrospective studies were excluded from this analysis, e.g., the IRIS study [38,39,40]. In case of overlapping publications emerging from the same study, the larger sample size study was evaluated, e.g., FALCON [41,42,43]. In case of additional information provided from multiple papers from the same trial, each article was evaluated separately.

### 2.2. Data Extraction

From each of the eligible studies, the following data were extracted: first author, year of publication, trial number, treatment arms, sample size, median age, median follow-up, progression-free survival (PFS), overall survival (OS), number of patients with the bone disease only, arthralgia rate, myalgia rate, bone pain incidence, back pain rate, osteoporosis and osteoporotic fracture rate. Two investigators, working independently, searched the literature and extracted data from each eligible study. Any differences were resolved via within-pair consensus.

## 3. Results

**Figure 1:**
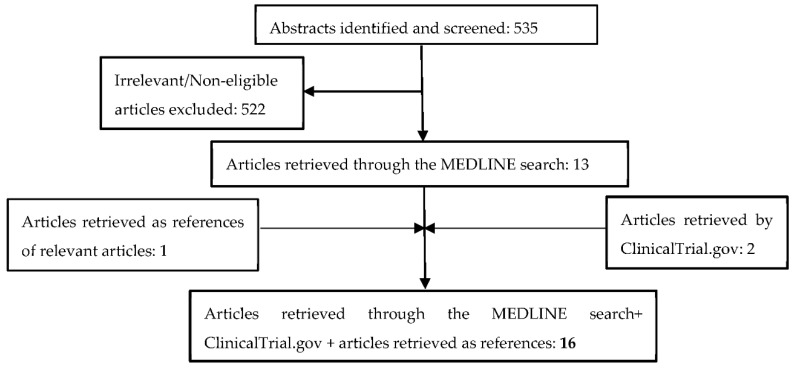
Consort flow diagram according to PRISMA 2009 guidelines.

Overall, 535 articles were identified and screened in MEDLINE Database. After the removal of irrelevant and non-eligible articles, 13 studies were considered eligible for our study [9,10,11,24,41,44,45,46,47,48,49,50,51]. After investigating the references of the eligible articles, one more study was added [52]. An additional search in ClinicalTrial.gov recruited two additional studies [53,54]. Overall, 16 studies were considered eligible for this systematic review. The aforementioned stages concerning the selection of studies are illustrated in Figure 1.

### 3.1. Arthralgia

In the metastatic setting, the reported percentage of arthralgia in patients under monotherapy with AIs ranged from 1 to 47% of patients treated with AI as monotherapy vs. 3.7–43% in patients receiving fulvestrant [41,44,46,47,48,49,50,51,52] (Table 1). The incidence of arthralgia in the population receiving the combination of fulvestrant plus AI was 12.1–44.5% [46,49,52]. The arthralgia rate reported in patients receiving exemestane was 5.6–47% [46,47,48,50], while anastrozole treatment-induced arthralgia in 1–45.1% of the cases [41,49,50,51,52].

In trials evaluating CDK4/6 inhibitor and AI combination treatment, arthralgia was reported in 5.8–33.3% of patients receiving the combination treatment [9,10,11,24,45,53,54] (Table 2). The arthralgia rates reported in patients receiving CDK4/6 inhibitors plus AIs tend to be lower compared with patients receiving AI monotherapy in the metastatic setting. In the MONALEESA-2 trial, the arthralgia rate was 27.5% in patients receiving ribociclib with AIs compared with the 28.8% arthralgia rate in the control arm [9]. This reduction in arthralgia incidence in patients receiving CDK4/6 inhibitors along with endocrine treatment was observed in MONARCHE-3 (12.8% vs. 16.7%) and MONARCHE PLUS trials as well (5.8% vs. 13.1%) [11,53]. Moreover, palbociclib demonstrated the highest arthralgia rate of the three CDK4/6 inhibitors (33.3%), while abemaciclib induced the lowest (5.8%–12.8%) [10,11,53,54]. Overall, the addition of CDK4/6 inhibitors seems to reduce the arthralgia events induced by monotherapy treatment with AIs.

### 3.2. Myalgia

In trials exploring AIs in metastatic disease, setting the incidence of myalgia reported was 2–23.7% in patients receiving AIs as monotherapy vs. 2–7% in fulvestrant monotherapy [41,46,47,49,50] (Table 1). Incidence of myalgias was 3.4–23.7% in patients treated with anastrozole [41,49,50] compared with 2–4.1% in patients treated with exemestane [46,47,50].

In Phase III trials exploring CDK4/6 inhibitors in the metastatic setting, myalgia rate was 4.8–11.9% in patients receiving CDK4/6 inhibitors and AIs [9,10,11,24,53,54] (Table 2). The incidence of myalgia was also decreased by the addition of CDK4/6 inhibitors to endocrine treatment, consistent with arthralgia rate.

### 3.3. Bone Pain

In the metastatic setting, bone pain was induced in 7–32.9% of patients treated with AIs monotherapy [46,48,49] (Table 1). Anastrozole induced bone pain in 27.8% of patients in the SWOG S0226 trial [49], while exemestane induced bone pain in 7–32.9% of patients in SOFEA and EORTC trials [46,48,55,56].

The addition of CDK4/6 inhibitors resulted in a 2.9–8.5% rate of bone pain as reported in Phase III trials [9,10,11,24,53] (Table 2). Treatment with palbociclib resulted more commonly in bone pain compared with ribociclib or abemaciclib (8.5% vs. 7.1–8% and 2.9–7.9%) [9,10,11,24,53]. Bone pain reported in patients receiving CDK4/6 inhibitors is decreased compared with patients treated with AIs for metastatic disease.

### 3.4. Back Pain

In metastatic disease, back pain was reported in 5.9–40% of patients treated with AI monotherapy vs. 1–10% of patients receiving fulvestrant [41,46,49,50,51,52] (Table 1). Anastrozole administration resulted in back pain in 5.9–40% of patients with metastatic breast cancer [41,49,50,51,52], whereas exemestane caused back pain in 7–11.4% of the same population [46,50].

CDK4/6 inhibitors caused back pain in 7.8–21.6% of patients in randomized Phase III trials [9,10,11,24,53,54] (Table 2). Consistent with the other musculoskeletal symptoms, back pain incidence was higher in patients receiving palbociclib (17.6–21.6%) compared with ribociclib or abemaciclib treated populations (18.5–20.6% and 7.8–12.2% respectively) [9,10,11,24,53,54]. In addition, back pain was more frequently reported in AI monotherapy treatment (5.9–40%) compared with CDK4/6 inhibitor and AI combination treatment (7.8–21.6%).

### 3.5. Osteoporosis-Osteoporotic Fracture

Osteoporosis incidence failed to reach the frequency threshold of 5% in trials of both AI monotherapy and CDK4/6 inhibitors in the metastatic setting [43,47,48,51,57,58,59,60,61].

Increased fracture susceptibility is considered as one of the most significant adverse events encountered in AI treatment. The incidence of fractures in AI monotherapy in the metastatic disease was rather low (0.2–0.6%) in the anastrozole-treated population, while there were no fractures reported in the exemestane arm [49,50,55,61] (Table 1). In trials exploring CDK4/6 inhibitors in combination with AIs, fracture rate was 0–0.45% in the treatment arm [9,10,24] (Table 2). Palbociclib in combination with letrozole resulted in a fracture in 0.45% of patients enrolled in the PALOMA-2 trial, while no fracture was reported in patients receiving ribociclib plus letrozole in MONALEESA-2 trial and only one patient presented with a fracture in the MONALEESA-7 trial [9,10,24,57,58]. No fractures were reported in patients receiving abemaciclib [11,59].

## 4. Discussion

Aromatase inhibitor-induced musculoskeletal syndrome (AIMSS) has emerged as a major cause of treatment discontinuation in hormone receptor-positive patients treated with AIs. In our study, arthralgia was reported in 1–47% of patients receiving aromatase inhibitors for metastatic breast cancer, while in patients treated with CDK4/6 inhibitors, the arthralgia rate dropped to 5.8–33.3%. According to our review of clinical trial data, it appears that CDK4/6 inhibitors may lead to the mitigation of AIMSS. However, it will be critical to collect more clinical trial data in the future to definitely prove this correlation. Crew et al. reported an incidence of 47% of joint pain in postmenopausal women treated with AI, including a 23.5% rate of new-onset joint pain and a 23.5% rate of exacerbation of preexisting arthralgia [66]. Moreover, joint stiffness was reported in 44% of the patients enrolled in the study. A recent meta-analysis exploring menopausal symptoms in postmenopausal women showed a 17.9% rate of arthralgia, ranging from 5.25 to 54.29% in the studies included [67]. Of note, the incidence of arthralgia was significantly decreased in early-stage breast cancer compared with advanced disease (RR = 0.34, 95% CI: 0.16−0.75) [67]. Overall, approximately 20–47% of women develop musculoskeletal symptoms during AI therapy, according to previous studies, which is consistent with our results. The upper and lower extremities, mainly hands, wrists, knees and ankles, seem to be the areas most affected by AI treatment [16]. Morales et al. tried to identify the radiological changes of AI-induced joint disease in breast cancer patients [68]. Ultrasound showed fluid accumulation in the tendon sheath surrounding the digital flexor tendons, and MRI demonstrated a thickening of the tendon sheath [68]. Between 6 months and two years, this intra-articular fluid further increased. Both steroidal and nonsteroidal aromatase inhibitors have been associated with AIMSS; however, nonsteroidal AI letrozole had a greater impact on bone mineral density (BMD) of the spine (*p* = 0.001) and hip (*p* = 0.075) than steroidal exemestane [69].

Given the high incidence of arthralgia in AI-treated breast cancer patients, the expected time to onset of symptoms should be identified. In an attempt to shed light on AI-induced musculoskeletal symptoms, a consortium on breast cancer pharmacogenomics (COBRA) trial was conducted in postmenopausal women with early breast cancer [16]. The median time to onset was 1.6 months (range 0.4–10 months); however, symptoms were reported as early as few days after treatment initiation. An observational study exploring the time course of treatment-related arthralgia reported consistent results (6 weeks until symptom initiation) and a gradual deterioration of symptom severity over 1 year (NCT00954564) [70]. Laroche et al. confirmed the development of joint pain after 6 weeks of treatment and that of a more diffuse pain after 12 months of treatment [71]. Henry et al. reported that the median time to treatment discontinuation due to toxicity was 6 months, while 25% of patients discontinued therapy due to musculoskeletal symptoms [14]. Collectively, the onset of symptoms occurs most commonly between two to three months from treatment initiation [16,68,70].

Three different mechanisms of AI-induced joint symptoms have been proposed [72]. The first and most likely one is based on estrogen deprivation caused by AI treatment. Aromatase inhibitors act by attenuating aromatase systematically even in peripheral tissues like breast and bone, where aromatase maintains local estrogen at reduced levels after menopause. It was shown that anastrozole decreased plasma levels of estrone, estradiol and estrone sulfate by a mean of 81%, 84.9% and 93.5%, while letrozole treatment decreased the same estrogen levels by 84.3%, 87.8% and 98.0% in postmenopausal women [73]. This decrease of serum estrogen concentrations induced by AI therapy leads to the repression of their bone-protective role within the joint. Estrogen depletion is also associated with an increase in local inflammation, even in the absence of systemic inflammatory markers like C-reactive protein and elevated sedimentation rate. Estrogen receptors, ERα and ERβ, are expressed in human articular chondrocytes and modulate the metabolism of chondrocytes [74]. Women are characterized by lower levels of ERα and ERβ receptors than men, and thus, they are more prone to cartilage erosion. The existence of estrogen receptors on human cartilage indicates the implication of estrogens in the joint microenvironment. Indeed, increased levels of ERα and ERβ have been identified in osteoarthritic joints, suggesting that chondrocytes respond to estrogen signals [75]. Furthermore, estrogens have direct effects on opioid pain fibers in the central nervous system and may affect pain transmission emerging from joint structures [76]. Pain is produced by joint structures innervated with nociceptive fibers like the periosteum, synovium and joint capsule. In the case of arthralgia, inflammatory mediators, including bradykinin and prostaglandins, are secreted to stimulate peripheral nociceptors so that they expand. In this way, nociceptive neurons become more sensitive to peripheral stimuli or central sensitization. Estrogens seem to implicate local inflammation and spinal transmission of pain, though the exact mechanism remains unknown [76]. However, estrogen receptors have been identified in opioid-containing neurons in the spinal cord and brain, further enhancing this notion [76]. Furthermore, it has been suggested that estrogens influence the spinal transmission of nociceptive stimuli through inhibition of microglial activation and inflammatory mediators, including prostaglandin E2 and nitric oxide synthase [75]. Animal studies and estrogen replacement therapy trials suggest a protective role of estrogens in osteoarthritis development [77,78]. Consistently, the high prevalence of osteoarthritis around the time of menopause indicates that estrogen depletion may contribute to arthritis development.

Another possible mechanism of treatment-induced musculoskeletal symptoms is through the induction of an autoimmune process that affects the joints, like rheumatoid arthritis [72]. Anastrozole increased the production of proinflammatory cytokines like IFN-γ, IL-12 and decreased IL-4 and IL-10 cytokine secretion in an animal model that simulates human rheumatoid arthritis [79,80]. Treatment with AIs stimulated an inflammatory response by activating CD4+ T cells and suppressing the differentiation of naïve T cells to Tregs that prevent autoimmune responses [79]. In this way, AIs promote the infiltration of the synovial membrane by CD4+ T cells and the cytokine-induced activation of monocytes, macrophages and fibroblasts in the joint microenvironment. Reduction of lymphocyte count, increased natural killer (NK) cell activation, and disruption of the IgG2a/IgG1 balance has been described during AI treatment [80]. Morel et al. described a case of a postmenopausal woman who presented with rheumatoid arthritis after four weeks of exemestane treatment [81]. Symmetric joint involvement, swelling of metacarpophalangeal and interphalangeal joints and increased inflammatory markers should raise the concern of autoimmune-related arthralgia. AIs have been linked to autoimmune diseases, including subacute cutaneous lupus erythematosus (SCLE), cutaneous vasculitis attributed to exemestane and deterioration of preexisting rheumatoid arthritis in a number of cases [80]. Moreover, discontinuation of AI treatment resulted in remission of arthralgia and a decrease in joint pain of over 50% [82]. Treatment discontinuation also led to the decrease of antinuclear antibodies (ANA) and rheumatoid factor (RF) autoimmune markers. These findings indicate the immunomodulatory effect of AIs as a potential mechanism of AIMSS in multiple cases. Finally, the third mechanism consists of a direct off-target effect of AIs or their metabolites, although this mechanism seems less likely.

Multiple factors influence the incidence of treatment-related arthralgia. High BMI and previous treatment with tamoxifen were shown to have a protective role against arthralgia occurrence [66,75]. On the other hand, certain chemotherapeutic drugs like taxanes and longer menopause duration have been associated with increased risk to the development of joint symptoms [14,66,71]. In addition, menopausal symptom severity and preexisting joint disease could be used as predictors of arthralgia development [70].

Most inflammatory processes, including arthritis, implicate the STAT3 (signal transducer and activator of transcription) and NFκΒ (nuclear factor κΒ) pathways. Tumor necrosis factor α (TNF-α), interleukin 1 (IL-1) and IL-6 activate STAT3 and NFκΒ pathways leading to epithelial cell activation and epithelial-mesenchymal transformation [83]. Estrogens inhibit IL-1 and IL-6-mediated bone absorption and stimulate OPG secretion, while estradiol inhibits osteoblast and stimulates osteoclast apoptosis via TGF-β [83]. Estrogen deficiency leads to elevated T-cell induced IL-6 and TNF-a, which are required for osteoclast generation [84]. Other proinflammatory cytokines regulating osteoclast production include IL-7, macrophage colony-stimulating factor (M-CSF) and most importantly, receptor activator of nuclear factor-kB ligand (RANKL), which is overexpressed in estrogen deficiency, leading to increased bone resorption. Estrogen loss leads to increased production of RANKL by stromal cells, increased activity of RANK and reduction of osteoprotegerin (OPG), promoting osteoclastogenesis. On the other hand, estrogen binding to ERα on osteoblasts leads to osteoprotegerin (OPG) production, which inhibits TGF-β and competitively inhibits RANK/RANKL interactions. As it is known, RANK/RANKL binding activates nuclear factor kappa B (NF-κΒ), resulting in the upregulation of the transcription factor nuclear factor of activated T-cells cytoplasmic 1 (NFATc1). NFATc1 catalyzes the maturation of osteoclast precursors to mature osteoclasts during osteoclastogenesis process.

How CDK4/6 inhibitors affect bone metabolism, and AI-related musculoskeletal symptoms is not clear yet. Cyclin-dependent kinases 4 and 6 (CDK4/6) regulate cell cycle progression via controlling the phosphorylation of retinoblastoma (RB) protein. In the hypophosphorylated state, RB represses the E2F family of transcription factors that regulate the passage in the S phase, acting as an onco-suppressor that blocks the cell cycle in the G1 phase. However, in response to mitogenic signals, CDK4/6 proteins form a complex with cyclin D, which catalyzes RB phosphorylation and reverses its repressive effect on E2F transcription factors [85]. Upregulation of CDK4/6—RB pathway is common in breast cancer, especially in luminal subtypes, where cyclin D1 amplification is identified in 58% of luminal B and 29% of luminal A cancers and CDK4 amplification in 25% and 14%, respectively [85]. In this way, CDK4/6 inhibitors inhibit aberrant cell proliferation by restoring the repressive effect of onco-suppressor RB protein on cell cycle progression. At the same time, CDK4/6 inhibitors affect other vital cellular activities. E2Fs consists of a family of transcriptional factors that bind target promoters and regulate gene expression. This family of transcriptional factors consists of both transcription-suppressors, such as E2F4, E2F5 and E2F6 and transcription activators like E2F1, E2F2, E2F3. It was shown that inhibition of E2F expression inhibited the proliferation of synovial cells and prevented cartilage invasion [86]. E2F2 is overexpressed in rheumatoid arthritis synovial tissues indicating a possible association between E2F2 and rheumatoid arthritis [87]. IL-6, TNF-α and LPS stimulate the expression of E2F2 in rheumatoid arthritis synovial fibroblasts via NF-κΒ, ERK and STAT3 pathway [87]. E2F2, in turn, activates the migration of synovial fibroblasts and the disease progression of RA. Hyperplasia and aberrant secretion of inflammatory factors in synovial fibroblasts leads to joint damage [86]. Another study demonstrated that E2F2 affects the STAT1 pathway and the subsequent activation of the PI3K/AKT/NF-κΒ pathway, which regulates the expression of IL-1α, IL-1β and TNF-α [86]. CDK4/6 inhibitors attenuate RB phosphorylation and preserve its suppressive effect on E2F transcriptional factors. Considering that arthritis and arthralgia are based on a complex inflammatory process, CDK4/6 inhibitors may attenuate E2F2 activity in synovium and cartilage and reverse, at least in part, the inflammation caused by aromatase inhibitors.

Recently, single nucleotide polymorphisms (SNPs) within genes controlling estrogen metabolism or signaling have been linked to AI-related arthralgia. SNPs in the CYP19A1 gene encoding the aromatase enzyme have been associated with an increased risk of arthralgia in patients treated with AIs. Park I.H. et al. associated a specific haplotype M_3_5 of the CYP19A1 gene composed of 16 SNPs with arthralgia incidence (*p* = 0.01) [88]. A subanalysis of the TEAM trial identified the homozygous variant rs934635 genotype of the CYP19A1 gene as a risk factor of musculoskeletal toxicity [89]. Other genetic variants of the CYP19A1 gene have also been associated with an increased incidence of arthralgia and bone loss after treatment with AIs [90,91]. In addition, Estrogen receptor 1 (ESR1) and ESR2 gene polymorphisms have been linked to treatment-related arthralgia [92]. Patients with ESR1 SNP rs2077647 variants exhibited lower rates of joint toxicity, while those with ESR2 SNP rs4986938 rare allele were at increased risk of arthralgia. Consistently, Henry et al. reported an increased discontinuation rate due to joint pain in patients with the genetic ESR1 variant rs9322336 [93]. Moreover, Garcia-Giralt et al. conducted a cohort study to evaluate the clinical role of SNPs within genes involved in estrogen and vitamin D metabolism. SNPs in steroid 17-alpha-hydroxylase/17,20 lyase (CYP17A1) and vitamin D3 receptor (VDR) genes were significantly associated with treatment-related arthralgia (*p* = 0.003 and *p* = 0.012, respectively) [94]. The clinical effect of these gene SNPs during treatment with CDK4/6 inhibitors is not yet characterized. The mitigation of AIMSS observed during CDK4/6 inhibitor administration may be partly attributed to currently unknown SNPs. Inversely, the SNPs previously described may predispose to the increased arthralgia observed in AI monotherapy.

## 5. Conlusions

We report an incidence of AI-induced musculoskeletal symptoms similar to the incidence described in previous studies and meta-analyses. The addition of CDK4/6 inhibitors in previous treatment with AIs tends to improve the incidence of this adverse event. More data from ongoing studies in the adjuvant and metastatic setting remain to clarify the effect of CDK4/6 inhibition on musculoskeletal symptoms.

## Figures and Tables

**Table 1:** Phase III randomized trials of aromatase inhibitors (AIs) in postmenopausal women with locally advanced or metastatic breast cancer.

Reference	Trial	Treatment Arms	Study Sample	Median Age	Median Follow-Up	PFS	OR	Bone Mets (%)	Arthralgia (%)	Myalgia (%)	Bone Pain (%)	Back Pain (%)	Osteoporosis (%)	Fracture (%)
**Johnston et al. 2013** [46]	**SOFEA** (NCT00253422, NCT00944918)	fulvestrant plus anastrozole vs. fulvestrant plus placebo vs. exemestane	723 (243/231/249)	63.8/63.4/66	37.9	4.4 vs. 4.8 (H: 1.0)	7% vs. 7% *p* = 0.88	15/16/13	40/43/47	4/2/2	9/6/7	7/10/7	NR	NR
						4.8 vs. 3.4 (H: 0.95)	7% vs. 4% *p* = 0. 27							
**Chia et al. 2007** [47]	**EFECT** (NCT00065325)	fulvestrant vs. exemestane	693 (351/342)	63/63	13	3.7/3.7 H: 0.93	7.4% vs. 6.7%; *p* = 0.73	67.2/66.4	3.7/5.6	4/4.1	NR	NR	NR	NR
**Robertson JFR et al. 2016** [41,43]	**FALCON** (NCT01602380)	fulvestrant vs. anastrozole	462 (230/232)	63.8/63.3		16.6 vs. 13.8; H: 0.797	46.1% vs. 44.9%; *p* = 0.729	10/10 (bone only)	16.6/10.3	7/3.4	NR	9.2/6	NR	NR
**Bergh J et al. 2012** [52,60]	**FACT** (NCT00256698)	fulvestrant plus anastrozole vs. anastrozole	514 (258/256)	65.2/63.4	8.9	10.8 vs. 10.2; H: 0.99	31.8 vs. 33.6; *p* = 0.76	63/71 (bone only)	12.1/10.6	NR	NR	5/5.9	NR	NR
**Paridaens RJ et al.** 200 [48,56]	**EORTC** (NCT00002777)	exemestane vs. tamoxifen	371 (182/189)	63/62	29	9.9 vs. 5.8; H:	46% vs. 31%; *p* = 0.005	35.1/35.4	11.5/5.3	NR	32.9/34.9	NR	NR	NR
**Mehta RS et al. 2012 et 2019** [49,55,62]	**SWOG TRIAL S0226** (NCT00075764)	anastrozole vs. anastrozole plus fulvestrant	694 (345/349)	65/65	84	13.5 vs. 15; H: 0.81	Median OS: 49.8 vs. 42; H: 0.82	22/21.5 (bone only)	45.1/44.5	23.7/22.9	27.8/32.7	40/38.2	NR	0.2/0.5
**Iwata et al. 2013** [50,61]	**NCT00143390**	exemestane vs. anastrozole	298 (149/149)	63.4/64	NR	13.8 vs. 11.1; H: 1.007	43.9% vs. 39.1%;	26.8/26.8 (bone only)	18.7/21.4	2.6/5.3	NR	11.4/14.7	NR	0/0.6
**Xu B et al. 2011** [51,63]	**NCT00327769**	fulvestrant vs. anastrozole	234 (121/113)	53.4/54.8		3.6 vs. 5.3; H: 1.31	10% vs. 14%; *p* = 0.343		4/1	NR	NR	1/6	NR	NR
**Howell A et al. 2005** [44]	**Trial 0020** **Trial 0021**	fulvestrant vs. anastrozole	851 (428/423)	63/63	27	5.5 vs. 4.1 (H, 0.95; *p* = 0.48)	27.4 vs. 27.7; (H: 0.98; *p* = 0.809)	19.9/19.6 (bone only)	8.3/12.8	NR	NR	NR	NR	NR

NR = not reported.

**Table 2:** Phase III randomized trials of CDK4/6 inhibitors in combination with AIs in postmenopausal women with advanced/metastatic breast cancer.

Reference	Trial	Treatment Arms	Study Sample	Median Age	Median Follow-Up	PFS	OR	Bone Mets Only	Arthralgia (%)	Myalgia (%)	Bone Pain (%)	Back Pain (%)	Osteoporosis (%)	Fracture (%)
**Richard S. Finn et al. 2016** [10,58]	**PALOMA-2** (NCT01740427)	palbociclib plus letrozole vs. letrozole plus placebo	666 (444/222)	62/61	23	24.8/14.5 (H: 0.58; *p* < 0.001)	42.1/34.7 (*p* = 0.06)	23.2/21.6	33.3/33.8	11.9/9	8.5/9	21.6/21.6	NR	0.45/0
**Martin et al. 2020** [45,64]	**PEARL** (NCT02028507)	**Cohort 1:** palbociclib plus exemestane vs. capecitabine **Cohort 2:** palbociclib plus fulvestrant vs. capecitabine	296 (153/143) 305 (149/156)	60/60 62/60	18.9 * 13.5	8 * vs. 10.6 (H: 1.11; *p* = 0.404) 7.5 vs. 10 (H: 1.13; *p* = 0.398)	NR	NR	NR	NR	NR	NR	NR	NR
**G.N. Hortobagyi et al. 2016** [9,57]	**MONALEESA-2** (NCT01958021)	ribociclib plus letrozole vs. placebo plus letrozole	668 (334/334)	62/63	26.4	25.3/16 (H: 0.568)	42.5/28.7; H: 0.746	20.7/23.4	27.5/28.8	6.5/6.3	7.1/10.6	20.6/17.8	NR	0/0.3
**Tripathy et al. 2018** [24,25]	**MONALEESA-7** (NCT02278120)	ribociclib plus tamoxifen/NSAI plus goserelin Vs placebo plus tamoxifen/NSAI plus goserelin	672 (335/337)	42.6/43.7	19.2	23.8 vs. 13 (H: 0·55, *p* < 0·0001)	NR	24.2/23.1	29.85/27.3	10.1/11	8/9.5	18.5/19.6	NR	0.3/0.3
**Matthew P. Goetz, 2017** [11,59]	**MONARCHE-3** (NCT02246621)	abemaciclib plus anastrozole or letrozole vs. placebo plus anastrozole or letrozole	493 (328/165)	63/63	26	Not reached vs. 14.7; H: 0.54	48.2/34.5	21.3/23.6	12.8/16.7	8.5/5.5	7.9/7.45	12.2/14.9	NR	NR
**NCT02763566** [53,65]	**MONARCHE PLUS**	abemaciclib plus NSAI vs. placebo plus NSAI or abemaciclib plus fulvestrant vs. placebo plus fulvestrant	463 (207/99/104/53)	56/59/55/58	26	Not reached vs. 14.7; H: 0.49	56/30 (*p* < 0.0001)	NR	5.8/13.1	4.8/5	2.9/5	7.8/9	NR	NR
						11.47 vs. 5.59; H: 0.37	38.5/7.5 (*p* < 0.0001)		6.7/5.6	1.9/0	0.9/1.8	4.8/5.6		
**NCT02600923** [54]		palbociclib plus letrozole	131	NR	NR	NR	NR	NR	20	6.1	NR	17.6	NR	NR

NR = not reported. * wild-type ESR1 population.

## Data Availability

All data can be found in MEDLINE bibliographical dataset.
